# Mechanical characterization of Bi-2212 composite winding pack samples for high-field superconducting magnet design

**DOI:** 10.1088/1361-6668/ae55d9

**Published:** 2026-04-01

**Authors:** Emma Martin, Youngjae Kim, Ulf Trociewitz, Daniel Davis, Aniket Ingrole, Shaon Barua, Eric Hellstrom, David Larbalestier, Fumitake Kametani

**Affiliations:** 1 Applied Superconductivity Center, National High Magnetic Field Laboratory, Florida State University, Tallahassee, FL 32310, United States of America; 2 Department of Mechanical and Aerospace Engineering, FAMU-FSU College of Engineering, Tallahassee, FL 32310, United States of America; 3 Magnet Science & Technology Division, National High Magnetic Field Laboratory, Florida State University, Tallahassee, FL 32310, United States of America

**Keywords:** Bi-2212 magnet, mechanical properties, mechanical reinforcement, composite magnet

## Abstract

Bi₂Sr₂CaCu₂O_8−*x*_ (Bi-2212) multi-filament round wire is a high-temperature superconductor (HTS) capable of carrying high transport currents, which makes it suitable for high-field magnet applications. However, its weak Ag–Mg sheath leaves it vulnerable to mechanical stress, posing challenges for high-field magnet design. To better understand and improve mechanical stress management in Bi-2212 winding packs, we conducted an experimental study evaluating the axial stress–strain behavior of five winding pack configurations with varying insulation materials, reinforcement strategies, and construction quality. Using uniaxial tensile testing at 77 K, we measured Young’s modulus and Poisson’s ratio for each composition. Our results show that pure alumina braid insulation and co-wind reinforcements significantly enhance stiffness compared to aluminosilicate braids, with more than 2.5 times increased winding pack Young’s modulus. Rule of mixtures analysis further quantified the contribution of non-wire composite components to overall stiffness. These findings highlight the critical role of insulation material selection and reinforcement design in optimizing Bi-2212 coil performance under stress, providing a foundation for improved mechanical models and more reliable high-field HTS magnet designs.

## 1. Introduction

Bi_2_Sr_2_CaCu_2_O_8−*x*_ (Bi-2212) multi-filament round wire is an isotropic, high-field capable, high temperature superconductor (HTS) that does not experience the large screening-current induced stresses suffered by HTS ReBCO coated conductors [1]. This conductor also possesses high current carrying capabilities of more than 1 kA mm^−2^, making it a very promising candidate for compact 20 + T high-homogeneity magnets [2]. However, superconducting coils tend to experience large mechanical stresses under operating conditions due to the Lorentz forces and a mismatch in the thermal contraction of different components [3, 4]. In a Bi-2212 28 T high homogeneity magnet design for a high-field NMR demonstrator magnet, insert coils will experience a peak stress of up to 700 MPa [5]. A significant challenge in the design of these magnets is understanding the mechanical properties of Bi-2212 windings and ensuring adequate mechanical stress management. Unlike ReBCO, which contains a high strength alloy substrate, Bi-2212 wire is sheathed with low strength Ag/Ag–Mg, leaving it susceptible to large stresses. These stresses, if left unmanaged, fracture the brittle superconducting filaments. Above 0.4% strain, the *I*_c_ of Bi-2212 begins to irreversibly decrease. By 0.6% strain, the *I*_c_ of the conductor will already be significantly degraded [6]. In an earlier study, the measured stress limit of a Bi-2212 strand reaching ∼0.6% strain was found to be 160 MPa [7]. The much higher stress anticipated in the high field NMR demonstrator project indicates the strong necessity of reinforcement and stress management techniques for the stable operation of Bi-2212 coils.

Our Bi-2212 magnets are designed to be operated at up to 0.4% strain. To delay the onset of performance degradation, our Bi-2212 wound magnets are reinforced so the magnets can withstand higher stresses before reaching the 0.4% strain threshold [8]. This is usually done by adding structural materials alongside the conductor during the coil fabrication process to create a composite winding pack. The Bi-2212 winding pack typically contains superconducting wire, electric insulation around the wire, reinforcement material, and an additional material to bond all components of the winding pack together. This bonding is usually done using epoxy in a vacuum-pressure impregnation process. Unfortunately, the addition of all these materials often results in a bulky magnet system with reduced winding current density. To balance out the need for mechanical reinforcement while maintaining high winding current density in Bi-2212 coils, computer modeling is often employed in the magnet design process. For computer models to yield accurate and useful results, it is important to understand the nature and properties of a Bi-2212 winding pack [9].

Understanding this composite winding pack is not straightforward as it contains several materials and material interfaces. Furthermore, the insulation surrounding the superconducting wire can be in the form of braided ceramic fiber. This ceramic fiber is used because it is compatible with the Bi-2212 over-pressure heat treatment (OPHT), which rises to 890 °C in a 1 bar O_2_ partial pressure environment. Additionally, the braid acts in a similar manner as filler material commonly used during the impregnation of other superconducting magnets [10]. The trajectories of the fibers (i.e. their tensile directions) in a braid are geometrically intricate and difficult to model. Hence, knowing the strength of the fiber does not necessarily translate to knowing the strength of the braid. For these reasons, it is challenging to model the winding pack or accurately establish its mechanical properties theoretically. Some studies, such as Li *et al*, have experimentally characterized the mechanical properties of a winding pack for magnet design purposes [9]. However, this study focused on a simple, unreinforced winding pack. Small changes in winding pack composition, such as variations in fill factor or the addition of reinforcement material, can have substantial impacts on the composite’s mechanical properties. In this paper, we describe an experimental study devised and conducted to aid magnet design by characterizing multiple winding pack compositions with varying insulation, reinforcement, and quality of construction. Here we report the Young’s modulus and Poisson’s ratio of five winding packs with varying reinforcement techniques and compositions.

## 2. Sample fabrication method

Braid-insulated, 1 mm diameter, Bi-2212 wire produced by Bruker-Oxford Superconducting Technology (B-OST) with 55 × 18 filament architecture was layer-wound around an Inconel racetrack-type mandrel into a typical hexagonal winding pack. The braiding material varied between samples and is detailed later. The windings then underwent a simple heat treatment at 1 bar atmospheric pressure in flowing pure oxygen. The windings were heated from room temperature to 600 °C at a rate of 200 °C h^−1^. They were kept at 600 °C for 2 h and then allowed to cool naturally to ambient temperature after the furnace was switched off. This heat treatment helped straighten the long sections of wire which aided in the fabrication of rectangular samples. The heat treatment also burnt off any sizing or coatings on fibers so they were in a similar condition as they would be after a full OPHT.

**Figure 1.**
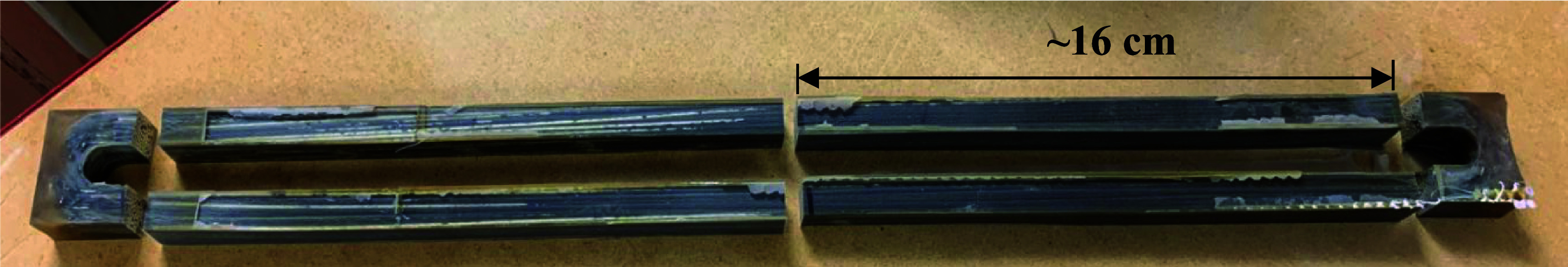
Photograph of impregnated windings showing cut locations to produce four, rectangular composite samples.

The windings were then transferred from the Inconel mandrel to a Teflon mandrel and impregnated with NHMFL-61, an epoxy mix developed at the National High Magnetic Field Laboratory (NHMFL), which is known to have high elongation before fracture and good thermal shock resistance [11]. Next, the impregnated windings were removed from the mandrel and cut into four individual rectangular samples for testing, as shown in figure 1. The samples had approximate dimensions of 1 cm × 2 cm × 16 cm. Five sample batches were fabricated with differences in the composition of each batch, giving 20 samples in total. However, the data set for Batch 4 only consists of three samples because of an issue that arose while testing one of the samples. The number of Bi-2212 wire strands in each sample varied between 80 and 100 depending on the winding pack design.

## 3. Sample specifications

Table 1 contains a summary of the sample descriptions. Batch 1 and 2 consisted of Bi-2212 wire coated with TiO_2_ and insulated with a 12-strand mullite braid [12]. The wire was coated in-house and then braided by B-OST. Mullite is an aluminosilicate typically consisting of 72% Al_2_O_3_ and 28% SiO_2_ by weight, with a reported elastic modulus of 131 GPa [13]. Each sample in these batches contained about 100 strands of Bi-2212. Batches 1 and 2 had random and hexagonal winding structures, respectively, as described later. Batch 3 also included hexagonally wound Bi-2212 wire coated with TiO_2_, insulated with the same mullite braid as Batches 1 and 2. However, these samples had an additional >99% purity alumina fiber co-wind. The alumina fiber has an elastic modulus of 370 GPa according to the manufacturer. We used a double co-wind, meaning two strands of co-wind were included for every strand of Bi-2212 wire, filling all interstitials between wires. Samples in Batch 3 also contained about 100 strands of Bi-2212 wire. Batches 4 and 5 consisted of hexagonally wound Bi-2212 wires that were without TiO_2_ coating but were insulated with an alumina braid. The wire was braided in-house using 12 strands of the same pure alumina fibers used for the co-wind in Batch 3. The samples in Batches 4 and 5 contained about 80 and 100 strands of Bi-2212 wire, respectively.

**Table 1.** Summary of sample descriptions.

Sample	Description	Wire volume fraction
Batch 1	Mullite braid, random wire arrangement	0.44
Batch 2	Mullite braid, hexagonal winding structure	0.37
Batch 3	Mullite braid, alumina co-wind, hexagonal winding structure	0.38
Batch 4	Alumina braid, hexagonal winding structure, voids	0.32
Batch 5	Alumina braid, hexagonal winding structure.	0.37

### 3.1. Measurement method

**Figure 2.**
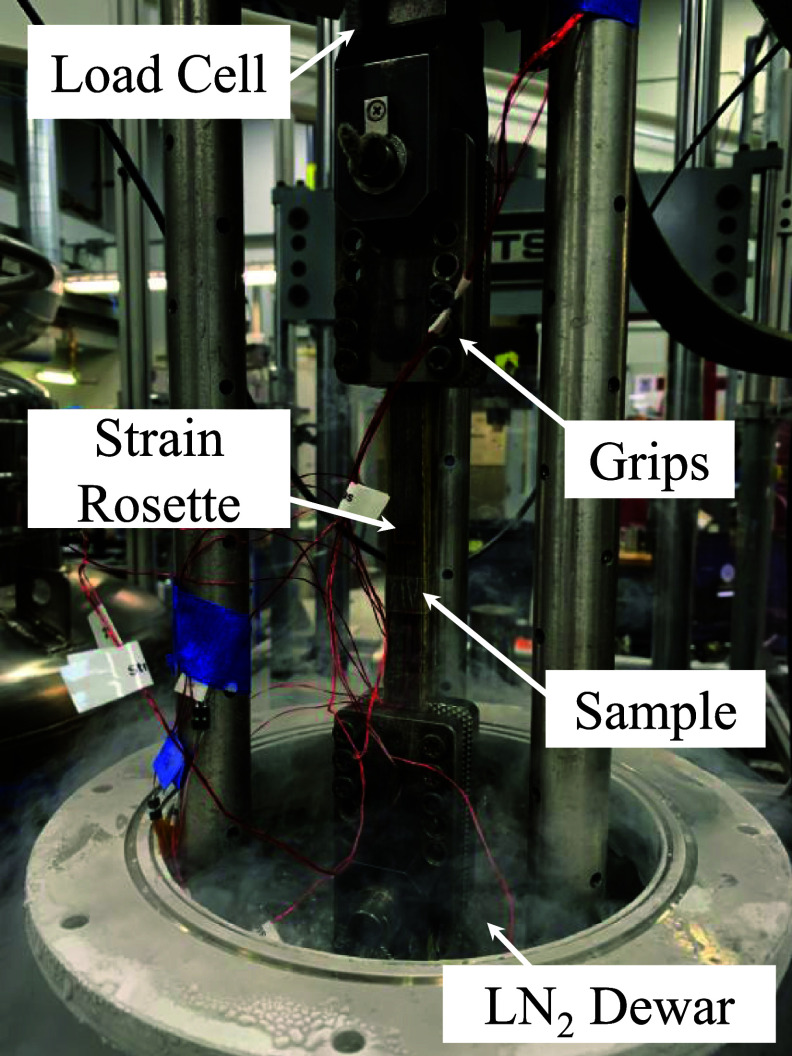
Tensile test set-up of Bi-2212 winding pack sample on MTS machine.

**Figure 3.**
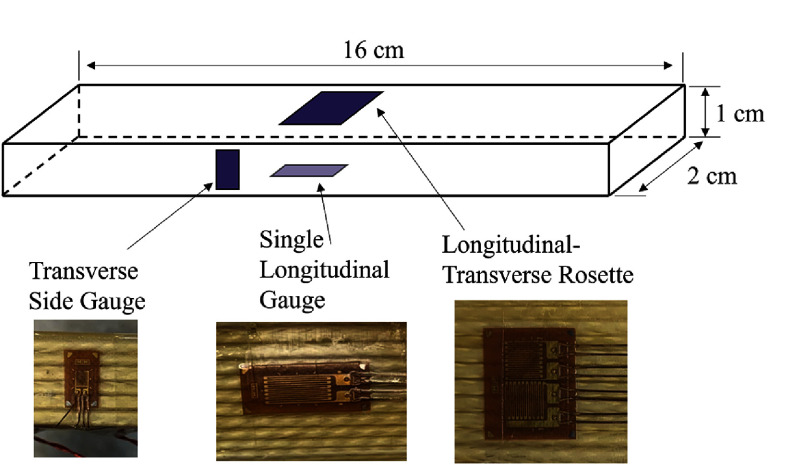
Sketch (top) of sample geometry outlining the strain gauge arrangement on samples with example images of each type (bottom).

Mechanical testing was done at 77 K at the NHMFL on an MTS servo-hydraulic test machine. Figure 2 shows how samples were clamped between grips and mounted in the 250 kN load cell for tensile testing. Figure 3 shows the combination of four strain gauges used for data collection and their locations on the samples. Two gauges were oriented in the axial direction and two in the transverse direction. Two gauges were placed in each direction to account for any bending stresses that may have occurred during testing. Stress–strain curves were recorded from uniaxial tensile tests with cyclic loading. Samples were placed in a cryostat, submerged in LN_2_, and strained at a rate of 0.5 mm min^−1^. The strain range of interest in this experiment was from 0%–0.4% strain, the usable range before the conductor typically experiences the onset of critical current degradation. Young’s modulus was calculated using Hooke’s law and the first loading cycle of each test.

## 4. Results

### 4.1. Structural features of samples

**Figure 4.**
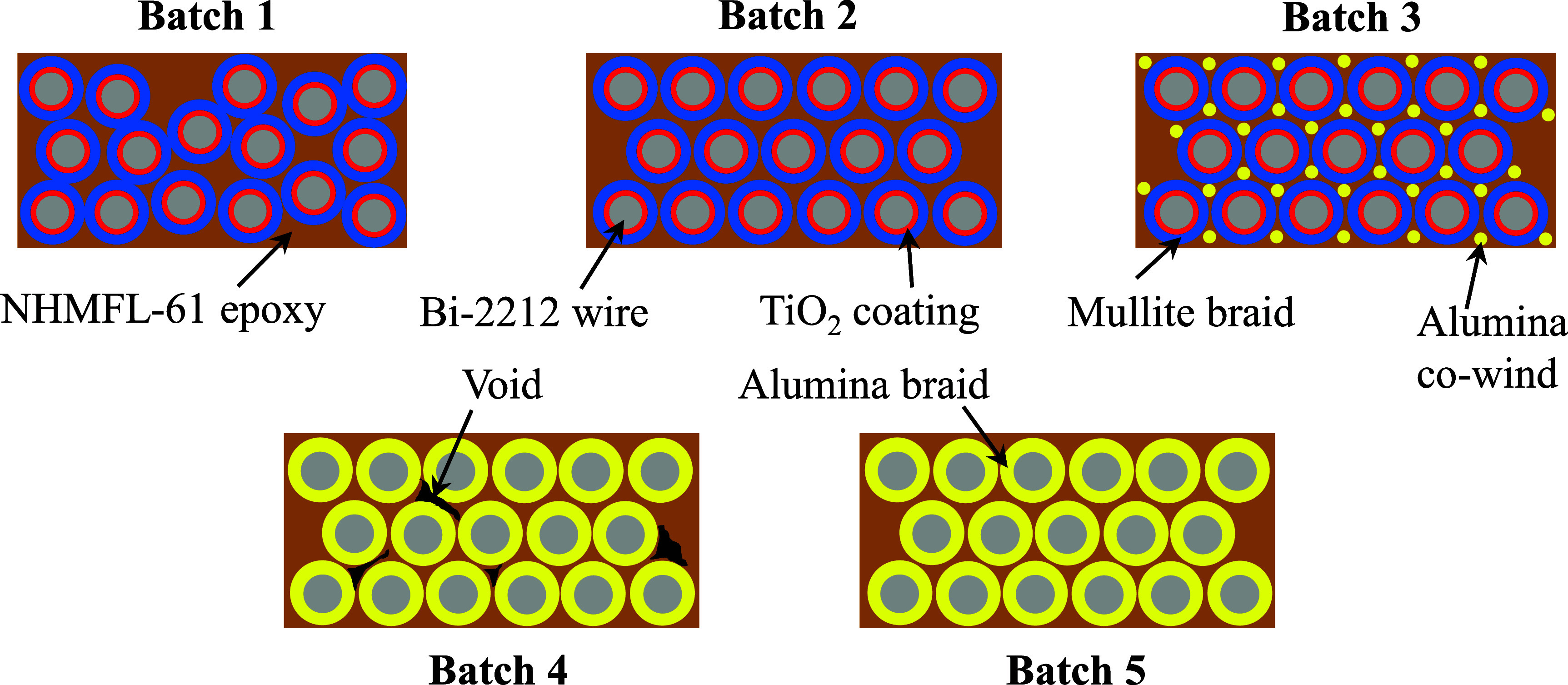
Schematic drawings showing the key structural features and materials used in each sample batch.

**Figure 5.**
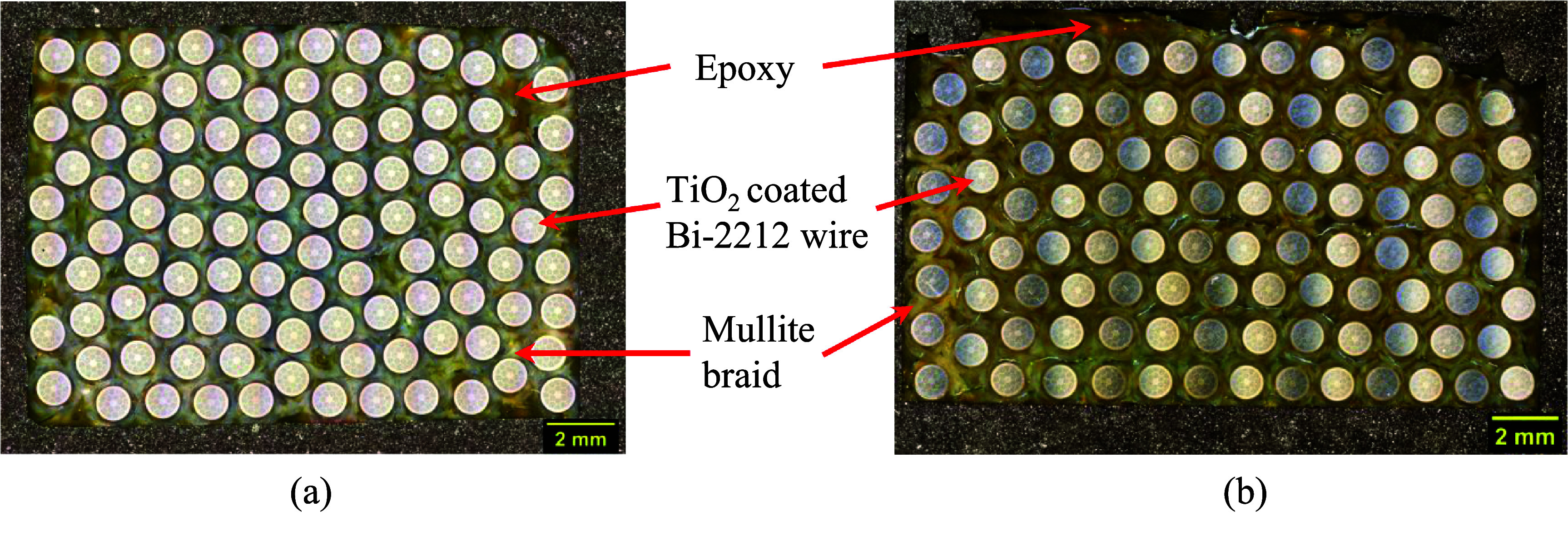
Cross-sectional images of winding pack samples from (a) Batch 1 and (b) Batch 2 showing the components of the composites.

Figure 4 displays schematic drawings summarizing the key features of each sample batch. Figure 5(a) shows a cross-sectional image of a Batch 1 sample showing the structural components (approximately 100 strands of Bi-2212 wire, epoxy, TiO_2_ coating and mullite). The TiO_2_ coated and braided wire had a total diameter of 1.28 mm. The wire alone occupied approximately 44% of the sample volume, with the remainder consisting of braided fibers and epoxy. As shown in figures 4 and 5(a), the Bi-2212 wires in Batch 1 did not maintain their hexagonal winding structure through the impregnation process and instead had a random arrangement and a slightly higher wire volume fraction than the other sample batches. Although this arrangement was not originally anticipated, we kept Batch 1 in our data set because these samples can still provide useful information about certain sections of coils. While most of the winding pack in a typical Bi-2212 coil has a hexagonal structure, there are usually some sections of the winding, such as the edges and near the terminals, that have a structure resembling Batch 1. Figure 5(b) shows a cross-sectional image of a Batch 2 sample. This batch had the same structural components as Batch 1 and also retained its hexagonal winding structure, as represented in figure 4. Batch 2 contained about 100 strands of Bi-2212 wire, occupying about 37% of the sample volume.

**Figure 6.**
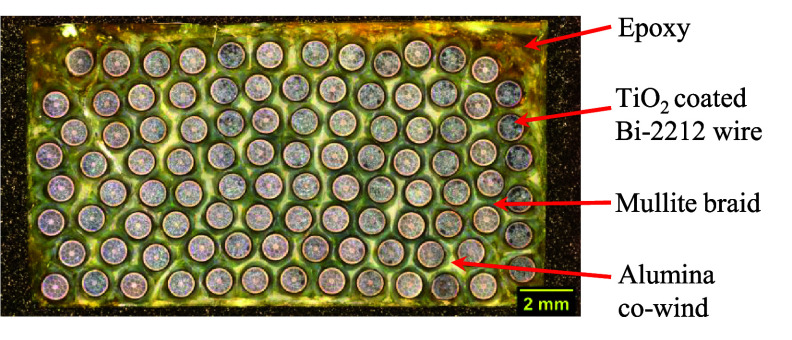
Cross-sectional image of winding pack sample from Batch 3 showinag the components of the composites.

Figure 6 shows a Batch 3 sample. The Bi-2212 wires in this batch also retained their hexagonal winding structure. In addition to the structural components included in Batches 1 and 2, this batch contained a >99% purity alumina fiber co-wind, which is represented in yellow in figure 4. The samples in this batch contained about 100 strands of Bi-2212 wire, which took up approximately 38% of the sample volume with the braided aluminosilicate, alumina strands, and epoxy making up the remainder of the sample.

**Figure 7.**
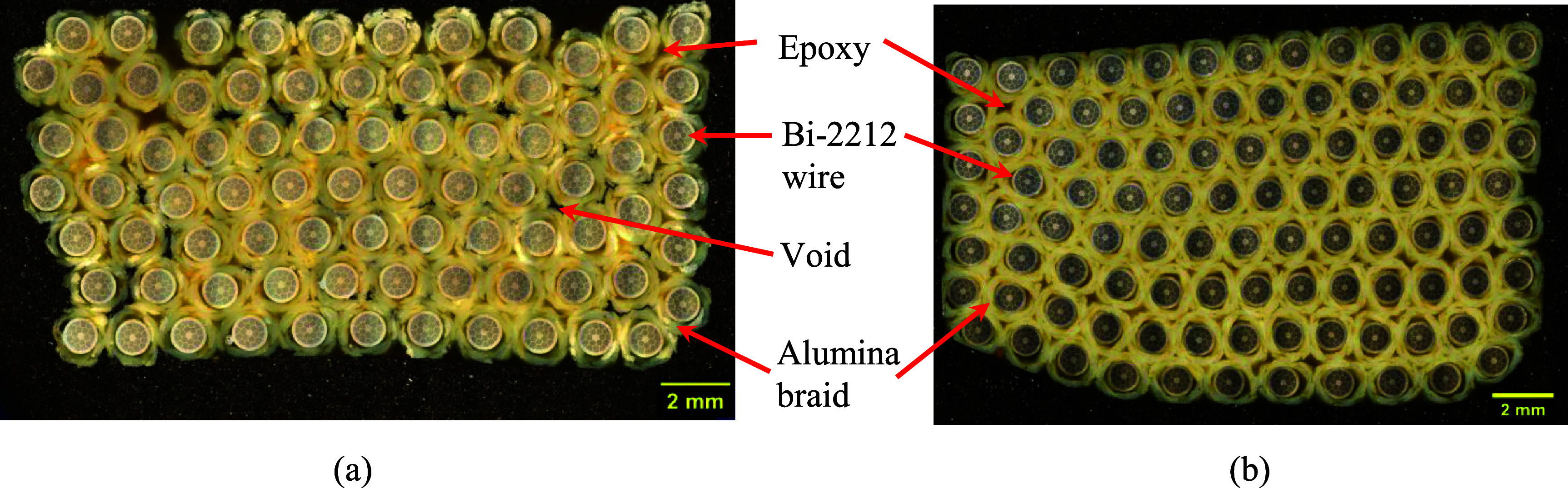
Cross-sectional images of winding pack samples from (a) Batch 4 and (b) Batch 5 showing the components of the composites.

Figure 7(a) shows the cross section of a Batch 4 sample consisting of hexagonally wound Bi-2212 wire. Figure 4 shows that unlike Batches 1–3, the 2212 wires were not coated with TiO_2_, but insulated with a 12-strand alumina braid. The alumina braided Bi-2212 wire had a larger diameter than the mullite braided wire, measuring 1.30 mm. Batch 4 samples also included voids distributed throughout the samples. The cross-sectional image shown in figure 7(a) was analyzed, revealing that the internal voids occupied less than 1% of the cross-sectional area. While it is possible to quantify the proportion of particular cross-sections that are taken up by void space, the voids were randomly distributed throughout the samples making it very difficult to extrapolate this value accurately for entire samples. Despite the voids, these samples were still valuable for this s**t**udy because voids sometimes occur in real coil winding packs and their consequences for the mechanical properties of coils need to be understood. These samples contained about 80 strands of Bi-2212 wire, accounting for approximately 32% of the sample volume, with the remainder consisting of braided alumina fibers, epoxy, and void space. As shown in figures 4 and 7(b), Batch 5 contained the same components and structure as Batch 4, but without void space. Batch 5 samples contained approximately 100 strands of superconducting wire, which occupied about 37% of the sample volume.

### 4.2. Elastic modulus measurements

**Figure 8.**
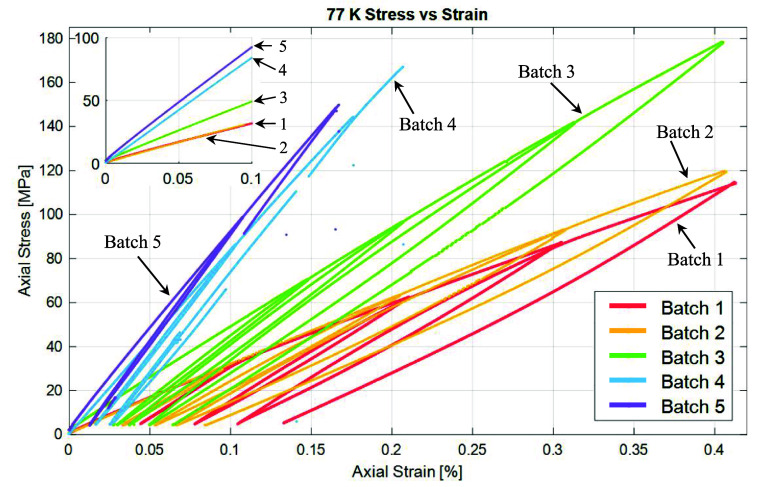
Axial stress vs strain curves for all sample compositions under cyclic loading at 77 K. The inset graph contains the first loading cycle of each curve, which was used to calculate Young’s modulus.

**Figure 9.**
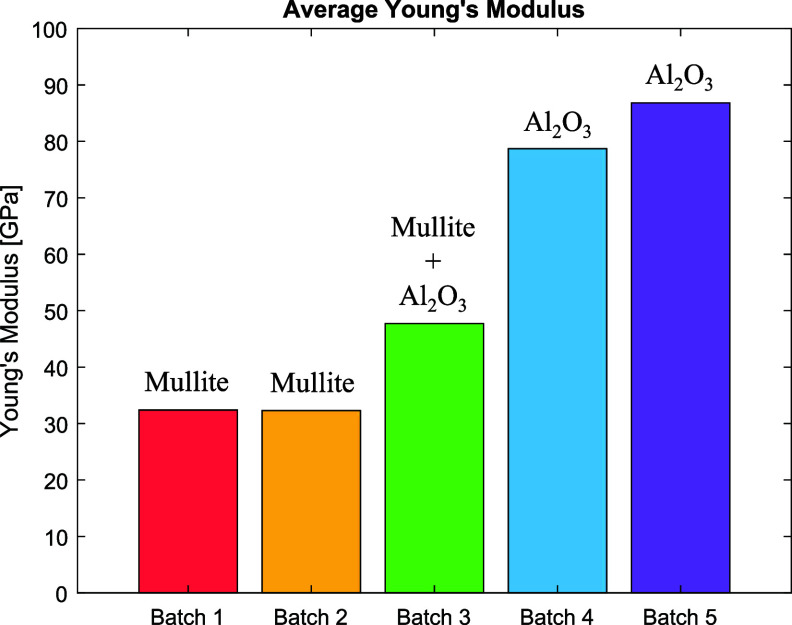
Bar chart showing the average Young’s modulus of each composite sample batch at 77 K.

Figure 8 shows the axial stress–strain curves for all sample types at 77 K from 0 to 0.4% strain. The first loading cycle of each curve was used to calculate Young’s modulus. This section of each curve is displayed in the inset graph of figure 8. Batches 1 and 2 show similar stress–strain curves despite having different wire arrangements in the winding structure and wire volume fractions. These batches are both composed of braided wire with no co-wind. However, as figure 5 and table 1 show, Batch 1 had a random wire arrangement with a higher wire volume fraction, while Batch 2 had a hexagonal wire arrangement and lower wire volume fraction. At very low strains, as seen in the inset graph of figure 8, the curves of the two batches are initially linear and almost identical. From these curves, the average Young’s modulus of Batch 1 was calculated to be 32.4 GPa, while the average modulus of Batch 2 was only 0.1 GPa different at 32.3 GPa. The Young’s moduli of the samples are represented visually in figure 9 and summarized in table 2. The standard deviation of each sample group is also shown in table 2, along with additional results that will be presented and discussed later. At higher strains, the stress–strain curves for Batches 1 and 2 do separate a little but maintain the same general shape: the slope decreases and curves slightly. These curves are fairly typical for fiber reinforced composites which consist of both a ductile, low strength matrix and brittle, higher strength fibers [14]. Samples from Batches 1 and 2 reach stresses of about 120 MPa at 0.4% strain. The similarity in the results from Batches 1 and 2 show that, provided the wire is running in the axial direction, and the epoxy performs as expected, the arrangement of the wires in the transverse cross section and small changes in the wire volume fraction do not have a large impact on the Young’s modulus of the entire winding pack.

**Table 2.** Experimental values for axial Young’s modulus and Poisson’s ratio of composite winding pack samples along with the standard deviation of the sample group. The axial Young’s modulus of the ‘matrix’ (all components of the composite minus the Bi-2212 wire) calculated using the rule of mixtures is also displayed.

Sample	Experimental Young’s modulus (GPa)	Calculated ‘matrix’ Young’s modulus (GPa)	Experimental Poisson’s ratio
Batch 1	32.4 ± 1.5	17.5	0.39 ± 0.04
Batch 2	32.3 ± 1.4	21.2	0.40 ± 0.04
Batch 3	47.6 ± 0.3	45.2	0.40 ± 0.02
Batch 4	78.7 ± 7.0	91.8	0.45 ± 0.03
Batch 5	86.8 ± 4.2	107.5	0.42 ± 0.03

Figure 8 also shows the stress–strain curves from the Batch 3 samples, which had the same general shape as Batches 1 and 2. However, the slope of the graph for Batch 3 is steeper than those of Batches 1 and 2. This batch was also braided with aluminosilicate fibers but included additional alumina co-wind fibers. Figure 9 shows that the average Young’s modulus of the Batch 3 samples was 47.6 GPa, almost 1.5 times greater than the moduli of Batches 1 and 2. This shows that the inclusion of an alumina co-wind increases the stiffness of the composite samples and further enhances their mechanical properties. Furthermore, the Batch 3 samples reached a stress of about 180 MPa at 0.4% strain, which is about 1.3 times greater stress than the Batch 1 and 2 samples at the same strain, showing that the inclusion of an alumina co-wind made the Batch 3 samples stronger and stiffer. It is worth noting that the co-wind in these samples did not reduce the density or number of Bi-2212 wires included in the sample since the alumina fibers were placed in the interstitials between Bi-2212 wires, as seen in the cross-sectional image in figure 6.

**Figure A1.**
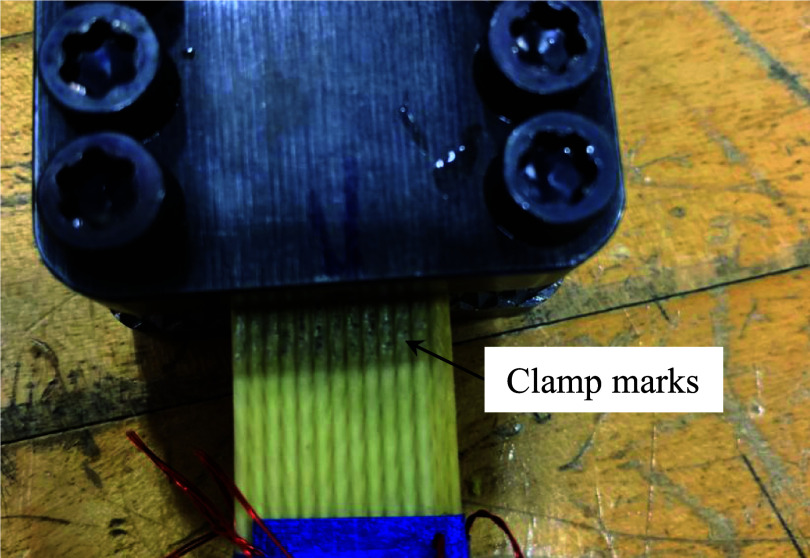
Photo of alumina braided sample after testing with clamping marks outside of the grips.

A significant finding in this study came from the testing of Batches 4 and 5 for which the braided fiber was alumina rather than aluminosilicate. Unfortunately, the tensile tests on Batches 4 and 5 could not be run all the way to 0.4% strain due to a clamping issue. The samples slipped in the grips during testing. Appendix figure A1 shows a photo of a sample with failed grips after testing. Figure 8 shows that unlike Batches 1, 2, and 3, the plots for Batches 4 and 5 remain linear and do not begin to curve at higher strains. This is presumably because these samples were not pushed to high enough strains for the nonlinearity to occur. The stress–strain plots for the alumina braided samples are much steeper than those of the aluminosilicate braided samples in Batches 1, 2, and 3, which contained the alumina fiber co-wind. Figure 9 and table 2 clearly demonstrate the higher stiffness of the alumina braided samples through the average Young’s modulus values displayed. The average Young’s modulus of Batch 4 was 78.7 GPa. This is about 1.6 times larger than that of the co-wound samples in Batch 3 and 2.4 times larger than the aluminosilicate braided samples in Batches 1 and 2, despite the presence of voids in Batch 4, which are shown in figure 7(a). Using the relationship between stress (*σ*), strain (*ϵ*), and Young’s modulus (*E*) stated in equation (1), it is possible to estimate the stress the Batch 4 samples may have experienced at 0.4% strain,

\begin{equation*}\sigma = E\varepsilon .\end{equation*}

Using the experimental Young’s modulus value stated in table 2, the Batch 4 samples could have theoretically experienced 315± 28 MPa of axial stress had they been tested up to 0.4% strain. This is far more stress than the Batch 1–3 samples experienced at that strain, and also larger than a Bi-2212 wire experiences at that strain in tension, which is around 140–150 MPa [8]. However, table 2 shows that the Batch 4 samples did have the largest variation in sample performance. This is most likely due to the random distribution of voids throughout the samples.

As expected, the void-free alumina braided samples in Batch 5 were stiffer than the samples containing voids in Batch 4, and the stiffest of all samples tested. Batch 5 samples had an average elastic modulus of 86.8 GPa, as seen in figure 9 and table 2. This modulus is 1.8 times higher than Batch 3, the aluminosilicate braided samples with the co-wind, and about 2.7 times higher than the aluminosilicate braided samples with no co-wind in Batches 1 and 2. Furthermore, using the relationship in equation (1) and the experimental Young’s modulus value in table 2, the Batch 5 samples may have theoretically experienced 347± 17 MPa of stress had they been tested to 0.4% strain. This is the largest stress of all the samples, and more than double the stress a Bi-2212 wire experiences at 0.4% strain [8]. We are working to resolve the clamping issue that prevented these samples from being tested all the way to 0.4% strain and are planning to fabricate and test additional alumina braided samples.

### 4.3. Rule of mixtures (ROM) calculations

Since all the samples did not contain the same number of Bi-2212 wires, the ROM was used to evaluate the mechanical properties of the non-2212 wire portion of the composites [15]. According to the ROM model and assuming uniform strain, equation (2) can be used to estimate the elastic modulus of a unidirectional composite with continuous parallel fibers:

\begin{equation*}{E_{\mathrm{c}}} = {E_{\mathrm{f}}}{v_{\mathrm{f}}} + {E_{\mathrm{m}}}\left( {1 - {v_{\mathrm{f}}}} \right)\end{equation*}

where *E*_c_, *E*_f_, and *E*_m_ are the elastic moduli of the composite, fibers, and matrix, respectively, and *v*_f_ is the volume fraction of the fibers in the composite [15]. Applying this equation to our series of winding pack samples, the Bi-2212 wires were considered the ‘fiber’ component as they run parallel to each other along the entire length of the samples. This left the remaining non-2212 portion in the samples, namely the epoxy, braid, and any additional reinforcement, as our ‘matrix’. Equation (2) was rearranged to solve for the elastic modulus of the matrix as seen in equation (3):

\begin{equation*}{E_{\mathrm{m}}} = \frac{{{E_{\mathrm{c}}} - { }{E_{2212{\text{ wire}}}}{v_{2212{\text{ wire}}}}}}{{\left( {1 - {v_{2212{\text{ wire}}}}} \right)}}.\end{equation*}

**Figure 10.**
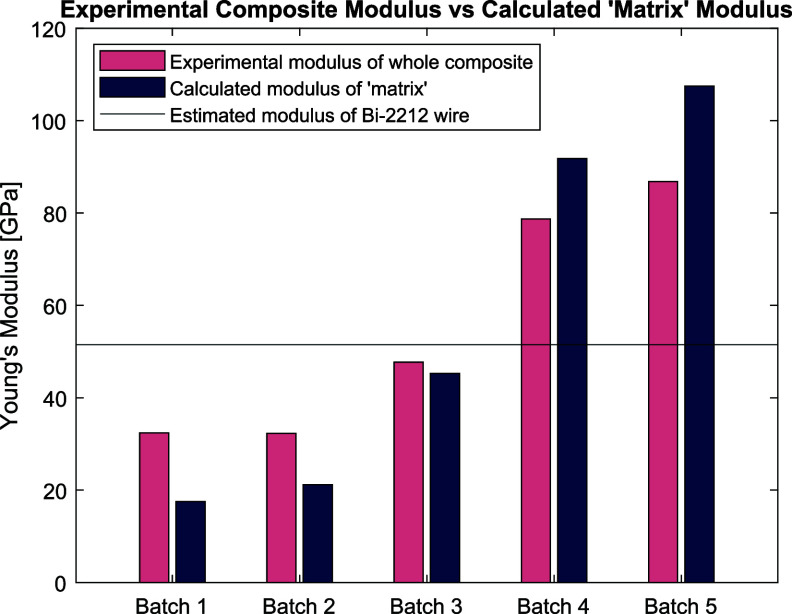
Bar chart showing the average measured Young’s modulus of whole samples for each batch, alongside the calculated ‘matrix’ Young’s modulus for each batch, and the estimated Young’s modulus of Bi-2212 wire used for the calculations.

The elastic modulus of the ‘matrix’ for each batch of samples was calculated using a combination of our experimental data and information found in literature. The experimental Young’s moduli displayed in table 2 were used as the values for *E*_c_ and the wire volume fractions shown in table 1 were used for *v*_2212 wire_. In a prior study, Mao *et al* reported the elastic modulus of a Bi-2212 wire reacted at 1 atm to be 51.5 GPa at 77 K, which we used as the value for *E*_2212 wire_ in our calculations [3]. The wire used in this study was assumed to have similar properties considering it also underwent a heat treatment at 1 atm and the samples were tested at 77 K. The results of these calculations can be seen in the bar chart in figure 10, where the calculated ‘matrix’ moduli are shown next to the measured moduli of whole samples for a visual comparison, as well as in table 2.

Figure 5 shows the ‘matrix’ of Batches 1 and 2 which consisted of aluminosilicate braid and epoxy surrounding the Bi-2212 wires. The ‘matrix’ elastic modulus of both these batches was around 20 GPa. This is lower than the experimentally measured modulus of these samples, as shown in figure 10, and less than half the value used for the elastic modulus of the Bi-2212 wire itself, 51.5 GPa. The low ‘matrix’ modulus values indicate that in these batches, the wire was the main source of reinforcement, not the braid or epoxy. However, it should be noted that the modulus of the ‘matrix’ in these samples was still considerably larger than that of the NHMFL-61 epoxy alone, which has been reported to be 0.7 GPa [11].

Batch 3 included aluminosilicate braid, epoxy, and an alumina co-wind between Bi-2212 wires for additional reinforcement, as seen in figure 6. Figure 10 shows that this ‘matrix’ was found to have an elastic modulus of 45.2 GPa, close to the experimental modulus of the entire Batch 3 samples. A comparison between the bars for Batches 1 and 2 and Batch 3 in figure 10 makes it clear that the inclusion of an alumina co-wind did increase the stiffness of the ‘matrix’. However, the modulus of the Batch 3 ‘matrix’ is still lower than the elastic modulus of a plain Bi-2212 wire, indicating that the wire is still the largest source of reinforcement in the Batch 3 samples, not the alumina fiber.

In contrast, the calculated ‘matrix’ modulus of Batches 4 and 5 was larger than the experimentally measured sample Young’s modulus values, as seen in figure 10. The ‘matrix’ of these samples consisted of alumina braided fibers and epoxy, with the addition of some void space in the case of Batch 4, as seen in figure 7. Batch 4’s ‘matrix’ modulus was calculated to be 91.8 GPa and Batch 5’s was 107.5 GPa. In the case of Batch 5, this is more than twice the elastic modulus of the Bi-2212 wire. These results show that the alumina braid significantly reinforces the ‘matrix’ (non-2212 wire portion) of the samples, so the composite’s stiffness is enhanced beyond that of Bi-2212 wires.

### 4.4. Poisson’s ratio measurements

**Figure 11.**
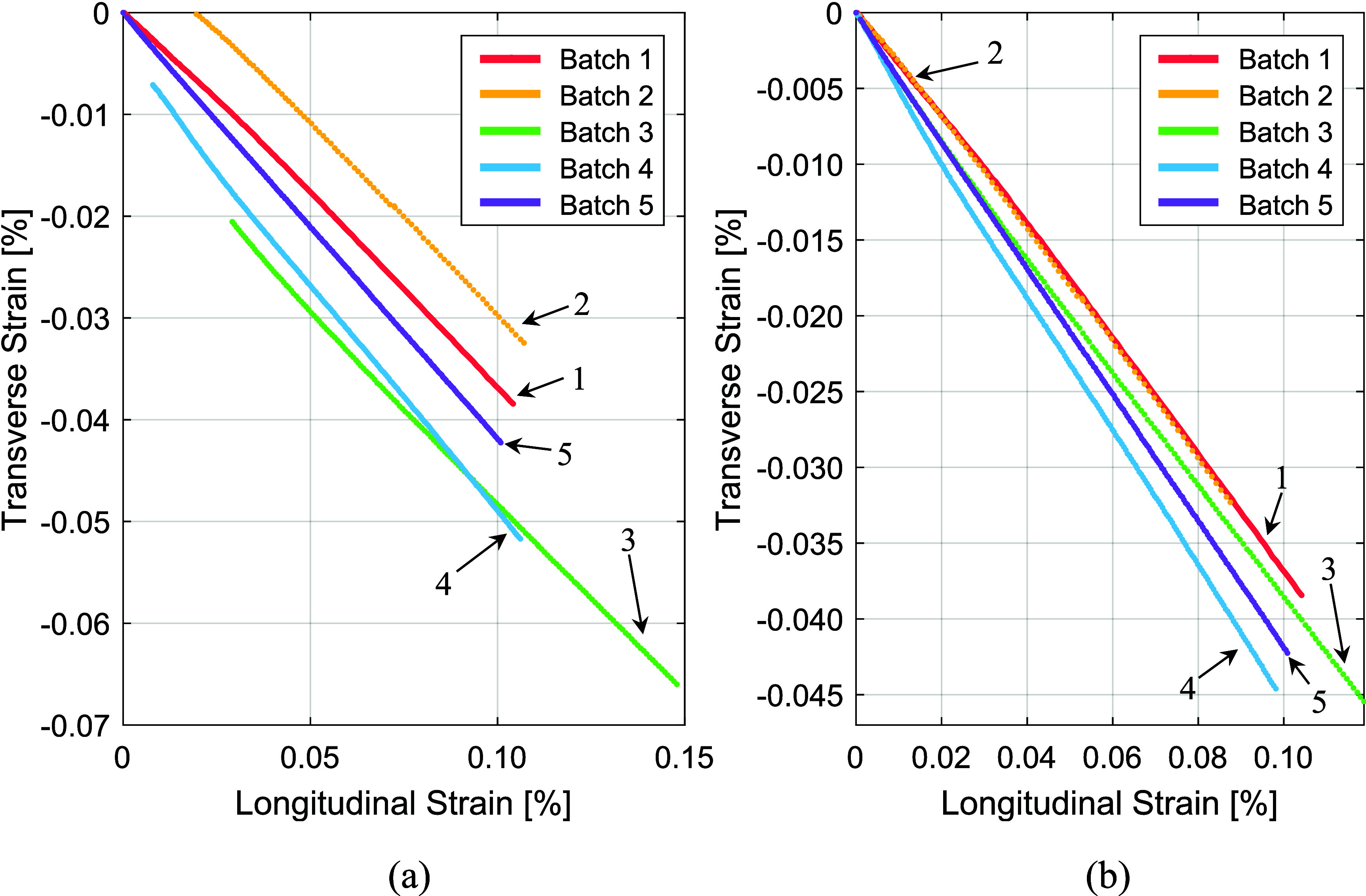
(a) Sections of transverse vs longitudinal strain curves at 77 K used to calculate Poisson’s ratios. (b) 77 K Transverse vs longitudinal strain curves offset to go through the origin.

As with the modulus measurements, Poisson’s ratios were calculated using the first loading cycle. The average value from each sample set, along with the standard deviation of the set, are displayed in table 2. Of the four samples tested in Batch 2, only two samples could be used to calculate the average Poisson’s ratio as the transverse strain gauges on the other two samples were damaged during testing. Figure 11 shows the transverse vs longitudinal strain graphs from representative samples. Figure 11(a) shows the section of the data used to calculate Poisson’s ratio. In figure 11(b) these data have been offset to go through the origin for an easy visual comparison of slopes.

At the low strains seen in figure 11, the samples all show linear behavior and have negative slopes. This indicates that the cross-sectional area of the samples decreased as they were axially lengthened, as expected. Batches 1, 2, and 3 all had average Poisson’s ratios of about 0.4. The alumina braided samples had slightly steeper slopes and higher Poisson’s ratios compared to the aluminosilicate braided samples. Batch 4’s average Poisson’s ratio was 0.45 and Batch 5’s was 0.42. Regardless of winding structure and reinforcement material, all samples generally had relatively high Poisson’s ratios. These large Poisson’s ratio values show that the samples all experienced substantial deformation in the transverse direction when stressed in the axial direction. This is not surprising considering the samples were bound together by soft epoxy and wires are surrounded by compressible fiber braids. Furthermore, the alumina braid was thicker than the aluminosilicate braid, so it follows that the alumina braided samples would be capable of larger compression in the transverse direction, and, therefore, have higher Poisson’s ratios. The largest Poisson’s ratio was seen in Batch 4, the alumina braided samples with voids. This is likely due to the voids in the sample allowing for greater contraction in the transverse direction during load cycles.

## 5. Discussion

Since the Bi-2212 winding pack is a composite material, its components and their arrangement within the composite significantly influence the mechanical properties of Bi-2212 coils. By changing the braiding and reinforcement materials, their configuration, and the amount included, we can drastically change the stiffness of the Bi-2212 winding pack. The five different Bi-2212 winding pack batches in this study provided us with several key technical insights into how to reinforce the Bi-2212 winding pack to manage the stresses induced by magnetic fields and during operation, which are discussed below.

Prior to this study, the contribution of the braid to the mechanical properties of the winding pack was unknown due to its complex geometry. Most fibers in the braid are not aligned in the axial direction and fibers crossing over each other create choke points. These complexities made it very difficult to implement the braid in our magnet models and simulations. During magnet design for simplicity of modeling, we conservatively assumed the elastic modulus of the braid to be the same as the epoxy, less than 1 GPa. Our results from the ROM calculations show that the braid contributes significantly more to the reinforcement of the winding pack than the epoxy. Even the aluminosilicate braided samples, which had the lowest elastic modulus of all the samples tested, had ‘matrix’ moduli of about 20 GPa, far higher than that of the epoxy. Furthermore, the ‘matrix’ calculations clearly showed that the alumina braided samples in Batches 4 and 5 had larger moduli than Bi-2212 wire, indicating that the alumina braid was the dominant component determining the stiffness in these samples. These results strongly suggest that the braid insulation is one of the two principal determinants for the stiffness of Bi-2212 winding packs, thus its contribution to the mechanical properties must be considered in the stress management design of Bi-2212 coils. The more accurate modulus values found in this study will serve as the foundation for our future FEM models.

Indeed, the stiffness of the Bi-2212 winding pack can be directly and significantly enhanced by using stiffer braid fiber. This is clearly demonstrated in the comparison between Batches 2 and 5. While the volume fraction of Bi-2212 wire was 37% in both batches, these two batches used different braid material. Aluminosilicate braid was used in Batch 2 and a much stiffer pure alumina braid in Batch 5, resulting in Batch 5 having a Young’s modulus more than 2.5 times larger than Batch 2. The effectiveness of alumina braid for stiffening the winding pack is an important finding for Bi-2212 coil R&D. The targeted application for these coils is in 25 + T magnet systems, where they will be exposed to, and must be able to withstand, very high magnetic stress.

We also evaluated the effectiveness of co-wind fibers on the mechanical properties of the Bi-2212 winding pack. Batch 3 was made to assess how co-wind fiber influences the strength and stiffness of the winding pack composite. Compared to Batches 1 and 2, which contained the same aluminosilicate braid but did not have co-winds, the Batch 3 samples exhibited 1.5 times greater Young’s modulus and withstood 1.3 times greater stress at 0.4% strain. These results indicate that the strength and stiffness of the winding pack can be further enhanced by adding co-wind fibers. An additional advantage of incorporating co-winds is their very marginal impact on winding density. As the co-wind fibers occupy existing interstitial spaces between wires, they do not compromise the overall packing efficiency, evidenced by the marginal difference in the wire volume fractions between Batches 2 and 3 (0.37 and 0.38, respectively). The addition of a co-wind means the interstitial spaces, which were originally filled with soft epoxy, were instead reinforced by tough alumina fibers, thus increasing the stiffness of the samples without reducing winding turn density. Furthermore, the amount of co-wind fibers can be adjusted, depending on the mechanical requirements of Bi-2212 coil designs. It is also possible to incorporate thicker co-wind fibers to increase the rigidity of winding pack while maintaining the current density of overall cross sections in the Bi-2212 coils.

When Bi-2212 wires are wound into a coil, it is often very difficult to maintain a uniform winding structure throughout the entire winding pack due to geometric constraints. Ideally, the layers of wound wires form a hexagonal packing arrangement as seen in Batches 2–5. However, certain sections, usually near the terminals or edges of the pack, often deviate from the ideal configuration. This raises a concern about whether such deviations of winding arrangement affect the mechanical properties of winding pack such as Young’s modulus and Poisson’s ratio. However, Batches 1 and 2, which exhibit markedly different transverse wire arrangements, and a variation in wire volume fraction, showed very similar Young’s modulus and Poisson’s ratio values. This suggests that a strictly hexagonal winding arrangement of Bi-2212 wires is not essential across all sections of the winding pack to preserve tensile Young’s modulus and Poisson’s ratio.

**Figure A2.**
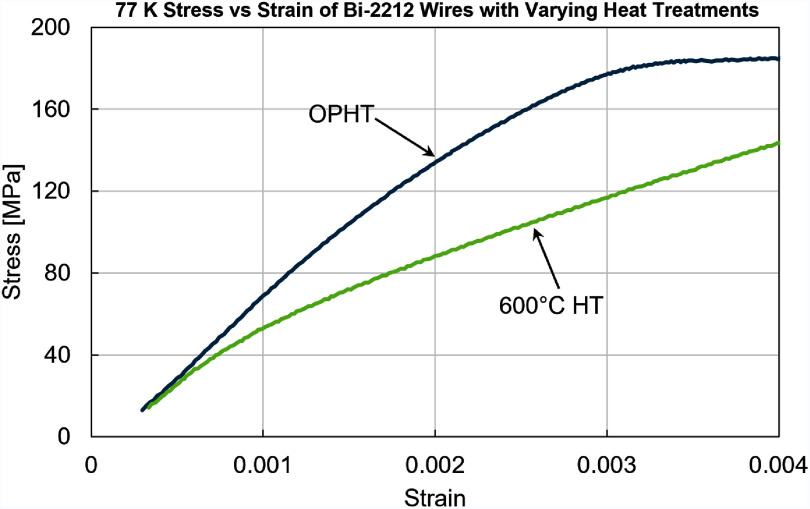
77 K Stress–strain curves for two pieces of pmm180929 Bi-2212 wire. One piece of the wire underwent a full OPHT before tensile testing. The other piece of wire underwent the 600 °C heat treatment described in this paper.

It is important to acknowledge that while the samples did undergo the simple 600 °C heat treatment described above, they did not undergo the full OPHT. This means that the Bi-2212 wire in the composite winding pack samples in this study does not have the same properties as Bi-2212 wire after the OPHT. Appendix figure A2 shows the 77 K stress strain curves for two pieces of the same Bi-2212 wire. One piece of the wire underwent a full OPHT while another piece of the same wire underwent the 600 °C heat treatment described in this paper. The 600 °C heat treated wire is less stiff and strong compared to the post-OPHT wire. This means that the experimental moduli reported in this paper are lower-bound estimates of fully functional Bi-2212 winding packs. Real Bi-2212 coils will likely show increased strength and stiffness compared to the composite winding pack samples tested here. The experimental moduli are useful as a direct comparison between different reinforcement techniques. They also serve as a good basis for conservative magnet models, while the ROM ‘matrix’ moduli can be used in conjunction with OPHT Bi-2212 wire mechanical properties for more robust modeling.

Our study emphasizes the need for a full evaluation of the matrix surrounding the Bi-2212 wires to understand the mechanical properties of a Bi-2212 coil winding pack. The properties of the matrix strongly depend on the insulation braid and co-wind fibers used. Therefore, correct materials selection and optimization of the structural arrangement of these components is essential. To realize the full high-field operation of Bi-2212 coils, it is also important to understand the thermal stress and strain incurred by the winding pack during cool-down for operation. The insulation braid and co-wind fibers have a large mismatch in thermal contraction compared to the Ag-sheathed Bi-2212 wires, potentially causing additional stress and strain which cannot be ignored during real coil operations. We are currently developing an experimental strategy to measure the thermal contraction of Bi-2212 winding packs. This will give us a more comprehensive understanding of the mechanical behavior of Bi-2212 coils during high field operations, in which the coils are exposed to the complex stresses and strains associated with large Lorentz forces and temperature changes.

Moreover, while this study focused on the axial mechanical properties of the winding pack, superconducting coils experience a complex combination of hoop tension, axial compression, and radial stresses. Thus, a comprehensive understanding of mechanical properties is essential for successful magnet design. In particular, the strength of the interfaces between the materials in the winding pack is very important. Under the high three-dimensional stress states in superconducting magnets, these interfaces may also be subjected to high stresses. Effective reinforcement of the superconductor relies on the assumption that stresses are transferred successfully between the constituent materials within the composite. Such stress transfer would not occur if the interfaces become de-bonded. Furthermore, other factors, such as the fatigue life of the winding pack must be well understood to ensure that magnets can survive their required lifespan. Therefore, the next step must be to measure inter-laminar shear, bending, and fatigue life.

## 6. Conclusion

The axial stress–strain behaviors of several Bi-2212 winding pack compositions were established experimentally, providing valuable mechanical property data for magnet design. Stress–strain measurements yielded more accurate Young’s modulus values and Poisson’s ratios for each composite, which are essential inputs for computer modeling. These refined parameters enhance the reliability of simulations used in high-field magnet design and performance prediction. The data clearly showed that incorporating an alumina co-wind significantly increases winding pack stiffness. Furthermore, changing the braiding material from mullite to alumina increased the Young’s modulus by more than 2.5 times. The rule of mixtures calculations reinforced that alumina, especially in braided form, is key to enabling Bi-2212 winding packs to withstand higher stresses while minimizing strain. The calculations revealed that the alumina braided composites were the only composites where the ‘matrix’ was stiffer than the Bi-2212 wire. In all the other composite batches, including Batch 3 which contained an alumina co-wind, the Bi-2212 wire was the main source of reinforcement, not the other components of the winding pack. By identifying which reinforcement techniques and materials produce a stiffer and stronger winding pack, this study provides a foundation for designing Bi-2212 magnets that are more mechanically robust and capable of reaching higher magnetic fields with improved structural integrity.

## Data Availability

Raw data collected as a part of this study can be found at: https://osf.io/6mvpy/overview.

## Appendix

Figure A1 displays a photograph of one of the alumina braided samples from this study after testing. The photograph shows that there are clamping marks outside of the grips. The presence of clamping marks outside of the grips indicates that the sample moved, or slipped, from its original position during the tensile test.

Figure A2 shows the stress–strain curves of two pieces of pmm180929 Bi-2212 wire at 77 K. The wires were tested in an MTS tensile test machine with a 5 kN load cell and were strained at a rate of 1 mm min^−1^. One of the wires underwent a full OPHT, while the other underwent the 1 atm 600 °C heat treatment described in this paper. The steeper curve of the wire after the OPHT indicates that it is stiffer than the wire that underwent the 600 °C heat treatment. Furthermore, at 0.4% strain, the post-OPHT wire experienced more stress than the 600 °C heat treated wire, around 180 MPa and 140 MPa, respectively, showing that in the region of interest, the post-OPHT wire is also stronger.
